# Effect of Membrane Surface Modification Using Chitosan Hydrochloride and Lactoferrin on the Properties of Astaxanthin-Loaded Liposomes

**DOI:** 10.3390/molecules25030610

**Published:** 2020-01-30

**Authors:** Mengdan Qiang, Xiao Pang, Dexue Ma, Cuicui Ma, Fuguo Liu

**Affiliations:** 1 Beijing Advanced Innovation Center for Food Nutrition and Human Health, Beijing Technology and Business University, Beijing 100048, China; 2 College of Food Science and Engineering, Northwest A&F University, Yangling 712100, Shaanxi, China

**Keywords:** astaxanthin, liposomes, membrane surface modification, chitosan hydrochloride, lactoferrin, stability

## Abstract

Astaxanthin-loaded liposomes were prepared by a thin-film ultrasonic method, and the effects of the different membrane surface modifiers chitosan hydrochloride (CH) and lactoferrin (LF) on the physicochemical stability of the liposomes and bioaccessibility of astaxanthin were studied. Based on the negative charge characteristics of egg yolk lecithin, LF and CH with positive charge were assembled on the surface of liposomes by an electrostatic deposition method. The optimal concentrations of modifiers were determined by particle size, zeta potential and encapsulation efficiency. The interaction between the liposomes and the coatings was characterized by Fourier Transform infrared spectroscopy. The stability of astaxanthin in different systems (suspension and liposomes) was investigated, and its antioxidant capacity and bioaccessibility were determined. The results showed that both membrane surface modifications could interact with liposomes and protect astaxanthin from oxidation or heat degradation and enhance the antioxidant activity of the liposome, therefore membrane surface modification played an important role in stabilizing the lipid bilayer. At the same time, the encapsulated astaxanthin exhibited higher in vitro bioaccessibility than the free astaxanthin. CH and LF modified liposomes can be developed as formulations for encapsulation and delivery of functional ingredients, providing a theoretical basis for the development of new astaxanthin series products.

## 1. Introduction

Astaxanthin is one of the non-vitamin carotenoids found in red yeast (*Haematococcus pluvialis*) and aquatic organisms such as salmon, trout and lobster. It mainly exists in free and esterified forms. The molecular structure of astaxanthin contains unsaturated double bond, which supplies the excellent antioxidant activity. Astaxanthin has been reported to have greater antioxidant activity than related carotenoids such as lutein, β-carotene, and lycopene [1,2]. As a nutraceutical, astaxanthin exhibits a variety of physiological activities, such as anti-oxidative stress and anti-inflammatory, immune response enhancing, cardiovascular disease preventing, motor function improving, etc., while also having a positive effect on both brain function and the central nervous system [3,4]. Therefore, if astaxanthin is added to food as a dietary functional ingredient, it will have practical significance for improving human health. However, due to the highly unsaturated molecular structure of astaxanthin, it is very sensitive to light, oxygen and temperature, which makes it easily to be oxidized, isomerized and degraded. At the same time, the poor solubility of astaxanthin in aqueous solution greatly reduces its bioavailability and limits its application in the food industry [5,6,7]. Studies have found that the use of delivery systems such as nanoparticles, emulsions, and liposomes is an effective way to improve the stability and bioavailability of poorly soluble bioactive ingredients [8,9]. As a nutrient carrier, liposome has good biocompatibility and can protect encapsulated nutrients from oxidation, improving their stability and bioavailability [10,11].

In recent years, increasing studies have confirmed that liposome encapsulation could improve the water solubility, antioxidant activity and cellular uptake of astaxanthin [12,13]. However, since liposomes are thermodynamically unstable, they may be susceptible to aggregation, fusion, and oxidation, leading to the destruction of their integrity, which in turn leads to the leakage of the encapsulated material. A practical approach to overcome these problems is to deposit biopolymers, including naturally occurring polysaccharides and proteins, onto the surface of the liposomes to maintain their structure and increase their kinetic and mechanical stability.

In this study, egg yolk lecithin and cholesterol were used as raw materials to prepare astaxanthin liposomes by a thin film ultrasonic method, then the liposomes were modified with chitosan hydrochloride (CH) and lactoferrin (LF). The physicochemical properties of the resulting liposomes, including particle size, polydispersity index (PdI), zeta potential, and encapsulation efficiency (EE) were investigated. The interaction between CH/LF and liposomes was further elucidated in detail via Fourier transform infrared (FTIR) spectroscopy. In addition, DPPH free radical scavenging ability, in vitro bioaccessibility, and liposome stability during storage under different environments were also evaluated.

## 2. Results and Discussion

### 2.1. Physicochemical Properties of Liposomes

The phosphatidylcholine in liposomes could be susceptible to hydrolysis under acidic conditions, and the alkaline environment is generally not suitable for food and pharmaceutical applications. Therefore, the astaxanthin liposomes were prepared at pH 7.0. At the same time, since both CH and LF are positively charged under neutral conditions (At pH 7.0, the zeta potentials of CH solution and LF solution are 46.97 ± 2.60 mV and 17.57 ± 0.58 mV, respectively), they could adsorb on the surface of negative-charged liposomes and thus improve the physical and chemical properties of the original liposomes.

#### 2.1.1. Particle Size

**Figure 1:**
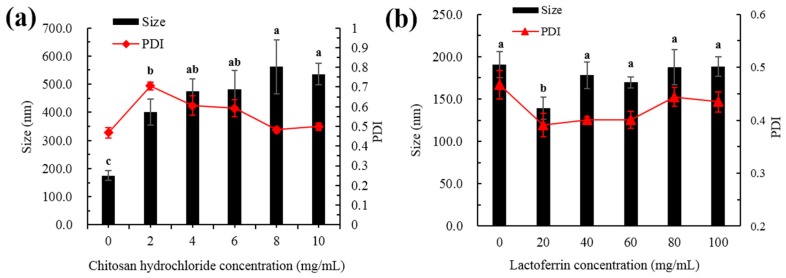
Particle size and PdI of astaxanthin liposomes at different concentrations of CH (**a**) and LF (**b**). Different lowercase letters on the column chart indicate significant differences between groups (*p* < 0.05).

The present study used five different concentrations of CH and LF to decorate the liposomes. As shown in Figure 1a, the bare liposomes exhibited a mean diameter of 191 ± 15 nm and a PdI of 0.47 ± 0.02. When the surface of the liposomes was decorated, the particle size gradually increased with the increase of CH concentration because CH interacts with the surface of the liposome membrane, and more CH was deposited on the surface of the liposome when the CH concentration increased. As shown in Figure 1b, with the increase of LF concentration, the particle size decreased first and then increased slowly. When the LF concentration was 20 mg/mL, the particle size decreased. This may be due to the instability of single liposomes, and aggregation between the particles was inhibited by the addition of LF, making the system more stable.

Modifications with appropriate concentrations are supposed to delay aggregation of the liposomes for prolonged periods of time. When the LF concentration was greater than 40 mg/mL, the particle size gradually increased. This may be due to the increase of film thickness caused by LF deposition on the film surface. Studies have also shown that membrane-modified liposomes exhibited better stability than unmodified liposomes [14,15].

PdI is a measure of the uniformity of particle sizes and can be used to reflect the homogenous size distribution of liposomes [16]. As shown in Figure 1a, the PdI value increased first and then decreased when the CH concentration increased. This was mainly because the liposomes were not completely modified with the low concentration CH, which made the particle size distribution non-uniform. With the increase of CH concentration, the surface of the membrane gradually became saturated, the particle size distribution of the whole system was uniform and eventually the PdI value decreased. As shown in Figure 1b, when the LF concentration was lower than 60 mg/mL, the PdI value decreased with the increasing protein concentration. The value of PdI was observed to increase at higher LF levels (80–100 mg/mL), while PdI values with all concentrations were smaller than that of the bare liposome. This indicated that the whole system was more uniform after the addition of LF.

#### 2.1.2. Zeta Potential

**Figure 2:**
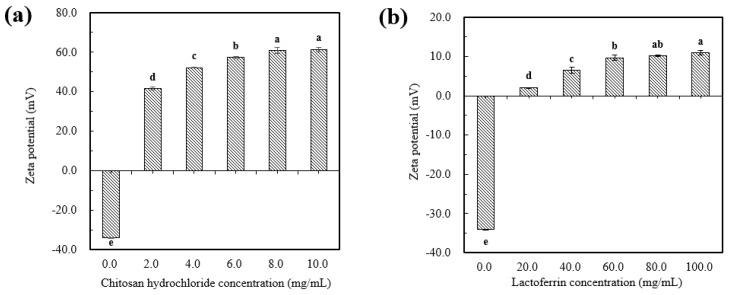
Zeta-potential of liposomes at different concentrations of CH (**a**) and LF (**b**). Different lowercase letters on the column chart indicate significant differences between groups (*p* < 0.05).

As shown in Figure 2a,b, the uncoated astaxanthin liposome (AST-Lip) was found to have a negative charge of −34.0 ± 1.3 mV, which might be due to the negatively charged phosphatidic acid and phosphate groups (PO_4_^3−^) present in the material. With increasing CH and LF concentrations, the zeta potentials of the astaxanthin-loaded liposomes modified with chitosan hydrochloride(CH-AST-Lip) and astaxanthin-loaded liposomes modified with lactoferrin (LF-AST-Lip) were both changed to positive after coating and gradually became saturated, which revealed that the positive chains of CH and LF adsorbed onto the surface of the membrane via electrostatic interaction, so the negative charges were neutralized and the zeta potential of the liposomes became positive. Furthermore, the values of the zeta potential of both CH-AST-Lip and LF-AST-Lip increased as their concentrations increased. The zeta-potential of CH-modified liposomes was higher compared to that of LF-modified liposomes, suggesting that CH-modified liposomes may have higher stability due to stronger electrostatic repulsion.

#### 2.1.3. Encapsulation Efficiency

**Figure 3:**
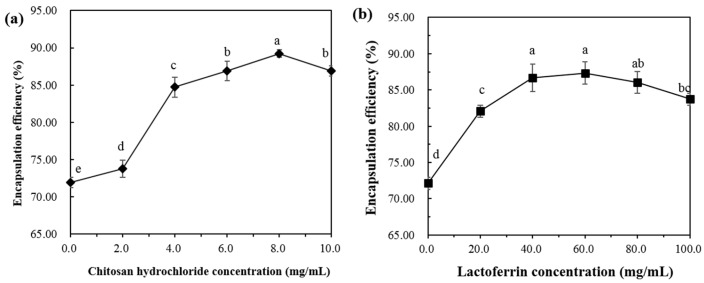
Encapsulation efficiency of liposomes at different concentrations of CH (**a**) and LF (**b**). Different lowercase letters indicate significant differences between groups (*p* < 0.05).

The encapsulation efficiency (EE) of astaxanthin in the lipid membrane is shown in Figure 3a,b. The EE of astaxanthin in the AST-Lip sample was 71.92 ± 0.68%. The relatively low EE is mainly due to the thin hydrophobic double layer of the liposomes. As shown in Figure 3a, with the increase of CH concentration, the EE increased with the increasing CH concentration from 0% to 8%, and became saturated. As shown in Figure 3b, the LF group exhibited the same trend as the CH sample, and the EE was the highest when the LF concentration reached 60 mg/mL. These phenomena were attributed to the fact that the added protein increased the rigidity of the liposomal bilayer and therefore reduced the leakage [17]. It has been reported that biopolymer coatings with proper concentration could increase the encapsulation ability for the core material of the liposome. For example, chitosan coatings around liposomes were found to inhibit the leakage of encapsulated compounds from the bilayer core [15]. Due to the electrostatic attraction, polymers could fill the gaps in the lipid via covering the surface of the liposomes [18,19], which would enormously reduce the leakage of astaxanthin. However, when the CH and LF increased to a certain extent, the EE values of the two groups showed a slight decrease. It can be inferred that a higher concentration of the modifiers may cause agglomeration of the vesicles, resulting in a lower EE.

Based on the results of particle size, zeta-potential and EE, the concentration of CH 8.0 mg/mL and LF 60.0 mg/mL was selected for the following experiments.

### 2.2. FTIR spectra Analysis

**Figure 4:**
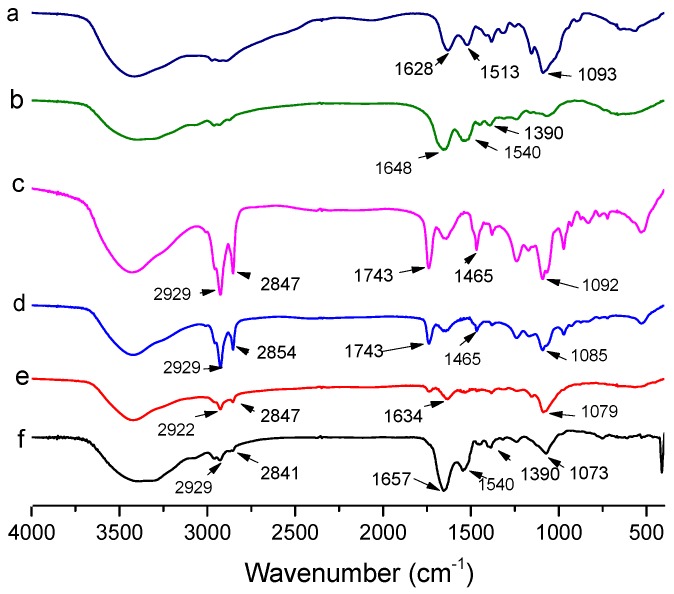
Fourier transform infrared spectra of different samples. a: CH, b: LF, c: blank liposome, d: AST-Lip, e: CH-AST-Lip and f: LF-AST-Lip.

The FTIR spectra of empty liposomes, CH, LF, AST-Lip, CH-AST-Lip and LF-AST-Lip were determined to explore the interaction among liposomes, CH and LF. As reported, the FTIR spectrum of astaxanthin showed characteristic absorption bands at 1648 cm^−1^ for the C=O stretching vibration, at 1552 cm^−1^ for the C=C stretching vibration, at 1074 cm^−1^ corresponded to C-O stretching vibrations and at 975 cm^−1^ corresponded to the C-H bending vibration in the C-C conjugate system [20]. Figure 4 shows the spectra and peak assignments for the different samples. Compared with the characteristic peaks of blank liposome, the spectra of astaxanthin liposomes in the absorption bands of 1074 and 975 cm^−1^ became weaker and shifted to lower wavenumbers, which demonstrated the possible interaction between astaxanthin and the lipid bilayer. LF exhibited absorption peaks of amide I and amide II bands at 1648 cm^−1^ and 1390 cm^−1^, respectively, and CH exhibited characteristic absorption peak at 1628 cm^−1^. But for LF-modified liposomes, the characteristic absorption peak of amide I shifted to 1657 cm^−1^. For CH-modified liposomes, the peak of CH-AST-Lip was shifted to 1634 cm^−1^, which suggested the interaction between coatings and liposomes, further confirming the binding between them.

### 2.3. DPPH Radical-Scavenging Activity

**Figure 5:**
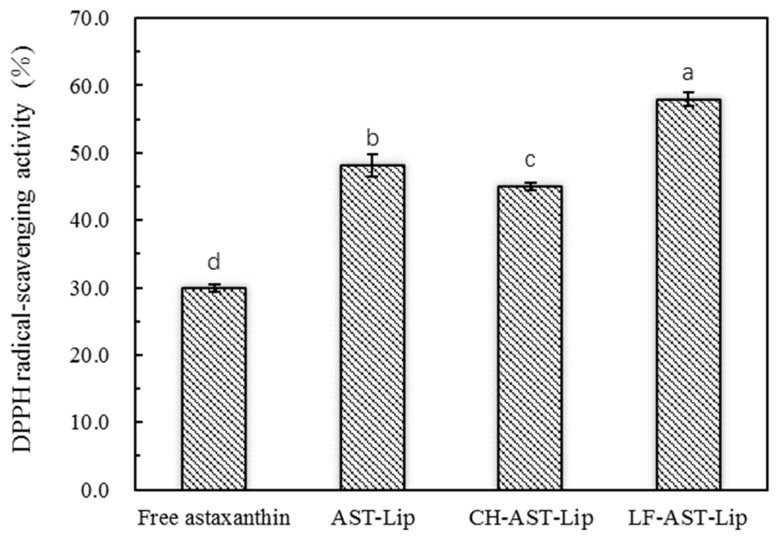
DPPH free radical scavenging rate of different samples, different lowercase letters on the column chart indicate significant differences between groups (*p* < 0.05).

DPPH· is a stable free radical that reacts directly with the hydrogen provided by the antioxidant to form a stable DPPH-H, which is extensively used to quantitatively determine the antioxidant capacity of biological samples. The single electron of DPPH radical has strong absorption at 517 nm. When a single electron is paired with a radical scavenger, the absorbance decreases and its color changes from purple to yellow. Since there are 11 conjugated double bonds and two β-ionone rings in astaxanthin, it has a strong DPPH· scavenging ability. As shown in Figure 5, AST-Lip showed higher DPPH· scavenging ability than astaxanthin in the suspension. This can be attributed to the fact that encapsulation may improve the dispersibility of astaxanthin, allowing it to be better dispersed in the system. The DPPH free radical scavenging rate of CH-AST-Lip was lower than that of AST-Lip, probably because CH had a certain protective effect on liposome membrane, and astaxanthin in CH-AST-Lip was not completely released. LF-AST-Lip exhibited the highest free radical scavenging rate, which might also depend on the antioxidant activity of LF itself.

### 2.4. Stability Evaluation

#### Thermal Stability and Storage Stability

**Figure 6:**
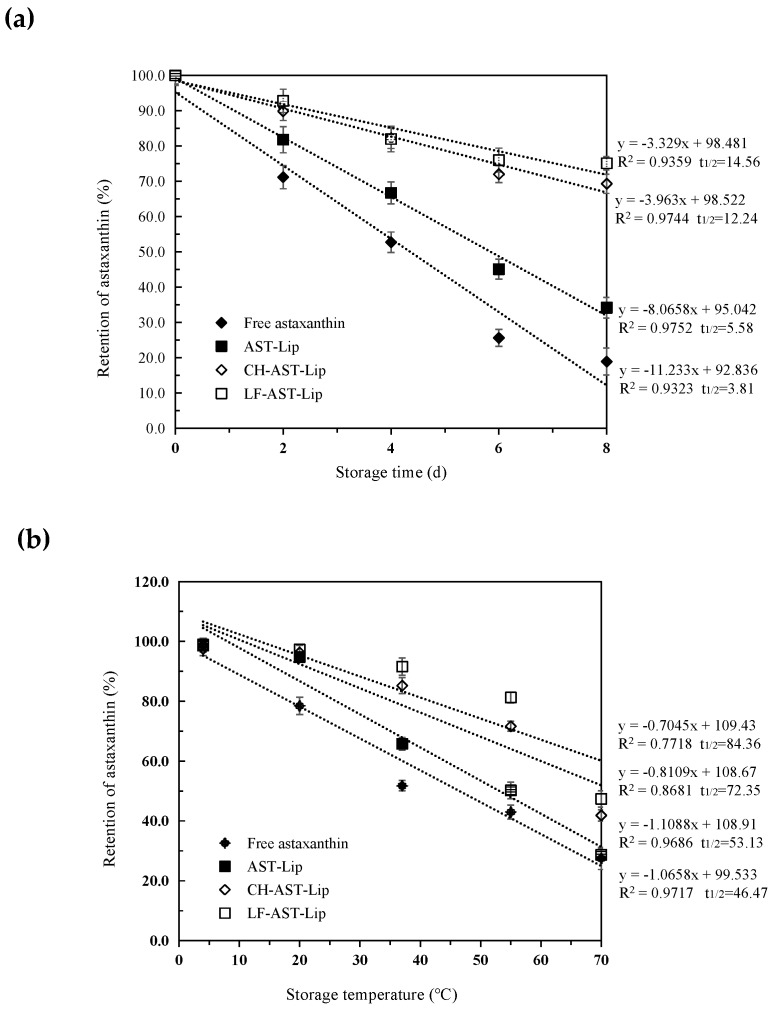
(**a**) Retention rate of astaxanthin in different samples under different storage time (**b**) Retention rate of astaxanthin in different samples under different storage temperatures.

Oxidation and heat can cause the degradation of astaxanthin during storage of the sample. Therefore, the effect of different media on the stability of astaxanthin was evaluated during storage. As shown in Figure 6a,b, the retention rate of astaxanthin against storage time and temperature revealed zero-order kinetic degradation characteristics. The rate constants *k* (slope of the degradation curve) and the t_1/2_ values (the time or temperature required to reduce astaxanthin retention to 50%) of astaxanthin were different for different samples, indicating that the stability of the encapsulated astaxanthin during storage was dependent on the type of the materials used to modify the liposomes. The retention of astaxanthin decreased in all samples during storage, and astaxanthin in the suspension exhibited the weakest stability. The t_1/2_ value of different samples increased in the following order: free astaxanthin< AST-Lip< CH-AST-Lip< LF-AST-Lip. The structure of the bare liposomes may be destroyed under high temperature, and CH and LF can be adsorbed on the surface of the liposome by the electrostatic action, thus effectively protecting the structure of the phospholipid bilayer membrane and avoiding the leakage and degradation of astaxanthin. LF-AST-Lip exhibited better stability than CH-AST-Lip, probably due to the stronger antioxidant capacity. In summary, liposome coatings, especially LF-modified coatings, could better protect astaxanthin from the damage caused by oxidation or heat.

### 2.5. Particle Size and Zeta-Potential of Different Liposomes during Storage

**Figure 7:**
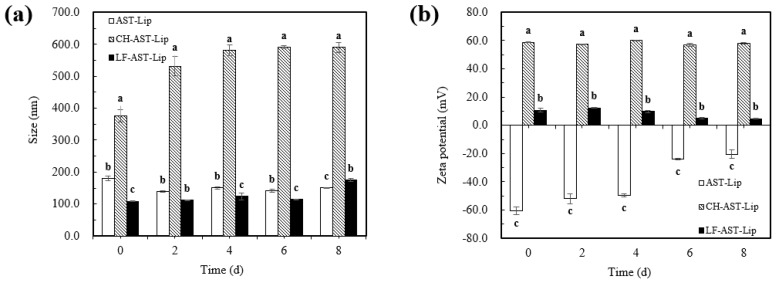
Particle size (**a**) and zeta potential (**b**) of samples during storage at 25 °C. Different lowercase letters indicate significant differences between groups (*p* < 0.05).

The physical stability of different liposomes was evaluated by measuring the change in particle size and charge of different samples during storage. Different liposomes were stored at 25 °C for storage experiments and it can be seen from Figure 7a that no significant changes in particle size of AST-Lip and LF-AST-Lip were observed, and the particle size was always less than 200 nm during storage, but the particle size of CH-AST-Lip increased significantly. The apparent increase in particle size of CH-AST-Lip might be via environmental factors such as temperature, which may change the structure of the polymerization network between CH and phospholipids. Agglomeration might generate large-sized particles in the system, resulting in an increase in particle size. Figure 7b reflected the change of zeta-potential of the samples during storage. Compared with the changes in zeta-potential of CH-AST-Lip and LF-AST-Lip, the zeta-potential of AST-Lip presented a more obvious change, which might be caused by that the phospholipid bilayer structure was destroyed, resulting in a decrease in the absolute value of the zeta-potential. Based on the above results, LF-modified liposomes showed better stability than other samples during short-term storage.

### 2.6. Bioaccessibility of Astaxanthin

**Figure 8:**
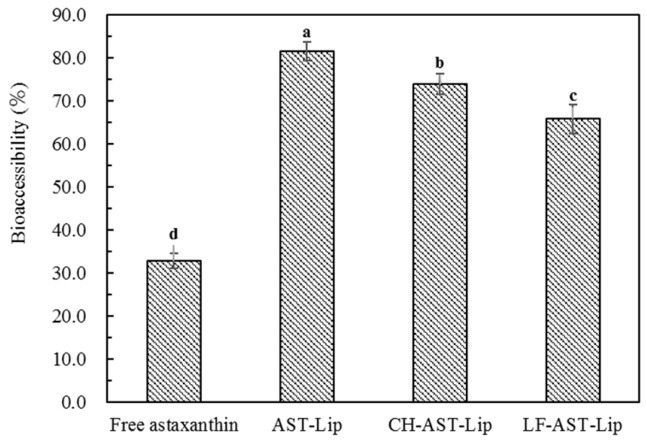
Bioaccessibility of different samples. Different lowercase letters indicate significant differences between groups (*p* < 0.05).

The in vitro digestion experiment was used to determine the bioaccessibility of astaxanthin. As shown in Figure 8, the bioaccessibility of astaxanthin in suspensions was low, probably because of the poor water solubility of astaxanthin, which is prone to aggregation during digestion. In addition, non-encapsulated astaxanthin cannot effectively transfer into the micelle phase during the digestion [11]. The bioaccessibility of AST-Lip, CH-AST-Lip, and LF-AST-Lip was higher than 65%, this was because liposome-loaded astaxanthin could greatly improve its dispersibility and thus enhance the bioaccessibility of astaxanthin. However, compared with AST-Lip, the bioaccessibility of astaxanthin in CH-AST-Lip and LF-AST-Lip was lower, which proved that the modification of CH and LF could improve the stability of the phospholipid bilayer, so the release of astaxanthin became sustained during the digestion time and less astaxanthin was effectively transferred into the micelle phases. In addition, the size of the liposomes may also affect their bioaccessibility, the large size of CH-AST-Lip resulted in small specific area, thus the rate of hydrolysis of lipids was reduced, resulting in lower bioaccessibility.

## 3. Materials and Methods

### 3.1. Materials

Astaxanthin (purity > 96%) were purchased from shanghai Yuanye Biotechnology Co., Ltd. (Shanghai, China). Egg yolk lecithin (PC-98T, purity > 98%) and cholesterol were obtained from A.V.T. Pharmaceutical Technology Co., Ltd. (Shanghai, China). CH (MW 50,000) was purchased from was purchased from Zhejiang Aoxing Biotechnology Co., Ltd. (Yuhuan, Zhejiang, China). Lactoferrin was bought from Westland Milk Products (Hokitika, New Zealand); DPPH (2,2-biphenyl-1-picrylhydrazyl, purity ≥97%) was from Aladdin Industrial Corporation (Shanghai, China).

### 3.2. Preparation of Astaxanthin Liposomes

The astaxanthin liposomes were prepared by a thin-film ultrasonic method according to the method of Pu et al. [18] with slight modifications. Briefly, Astaxanthin, lecithin, and cholesterol were dissolved together in 240 mL chloroform with concentrations of 0.2 mg/mL, 4 mg/mL and 0.4 mg/mL, respectively. Then the chloroform was removed by vacuum evaporation at 55 °C using a rotary evaporator (Shanghai Kusnc Co., Ltd., Shanghai, China), and the sample was further subjected to vacuum drying to ensure complete elimination of the organic solvent. Then, the formed films were dispersed in aqueous solution (pH 7.0) and sonicated for 10 min at 180 W in an ice-cold water bath with a cycle of 2 s sonication and 2 s standing. The prepared astaxanthin liposomes (AST-Lip) was sealed with nitrogen and stored at 4 °C in the dark.

### 3.3. Preparation of CH and LF Decorated Astaxanthin Liposomes

The astaxanthin liposomes modified by CH and LF were prepared as follows: 10 mL solution containing CH or LF was dissolved in the aqueous solution (pH 7.0). The astaxanthin liposomes were added dropwise to the solution containing an equal volume of CH or LF and stirred with a magnetic stirrer at 800 r/min for 2 h. The final concentrations of CH were set at 2, 4, 6, 8, and 10 mg/mL. The final concentrations of LF were set at 20, 40, 60, 80 and 100 mg/mL. The prepared CH-astaxanthin liposomes (CH-AST-Lip) and LF-astaxanthin liposomes (LF-AST-Lip) were stored at 4 °C in the dark. The mixture of astaxanthin suspension and blank liposomes was prepared as the control group. The astaxanthin suspension was prepared by dissolving astaxanthin in dimethyl sulfoxide (DMSO, Sinopharm Chemical Reagent Beijing Co., Ltd., Beijing, China). The concentration of astaxanthin in the suspension was consistent with the astaxanthin liposomes. Then a certain amount of the astaxanthin suspension was mixed with the blank liposomes.

### 3.4. Measurement of Encapsulation Efficiency (EE)

The content of astaxanthin in the liposomes was measured by referring to a standard curve prepared as follows: Astaxanthin standard solution was prepared by dissolving astaxanthin sample in chloroform, in which chloroform was used as a blank. Then the absorbance at 473 nm was detected utilizing an ultraviolet (UV)–visible spectrophotometer (Shimadzu UV-1240, Tokyo, Japan). The standard calibration curve of astaxanthin was obtained (y = 76.186x + 0.0676, R^2^ = 0.998). The EE of astaxanthin in the sample was measured by centrifugal extraction and chemical colorimetry [12]. Briefly, 400 μL of astaxanthin liposomes and 5 mL of petroleum ether were gently mixed and stirred at 30 °C for 5 min, the mixture was centrifuged at 3000 rpm for 5 min, and the supernatant was collected. The above steps were repeated twice. All collected supernatants were then transferred to a centrifuge tube and the petroleum ether was removed using a nitrogen blow dryer. Finally, the residue obtained after evaporation was dissolved in chloroform, with the content of free astaxanthin determined at 473 nm. Each experiment was conducted in triplicate. The astaxanthin EE (%) was calculated using the following Equation (1):

(1) $$EE=\left[\frac{\left(m_{1}\;-\;m_{2}\right)}{m_{1}}\right]\times 100\%,$$

where EE is the encapsulation efficiency of astaxanthin, m_1_ is the total amount of initial astaxanthin added, m_2_ is the amount of unencapsulated astaxanthin.

### 3.5. Determination of Size and Zeta Potential

The size and zeta potential of the liposomes were determined using a Malvern Zetasizer ZS 3600 (Malvern Instruments, Worcestershire, UK) at 25 °C with a scattering angle of 90°. To avoid multiple scattering effects, the liposomal suspension was diluted 15-fold with the distilled water before the determination.

The diluted sample was added to the sample cell and the particle diameters of the different samples were measured. The results were expressed by the mean diameter (nm) and polydispersity index (PdI). The measurements were repeated three times.

The diluted sample was added to a potential cell and the zeta potential of the sample was determined by measuring the direction and velocity of droplet movement in a defined electric field. The measurements were repeated three times.

### 3.6. Fourier Transform Infrared (FTIR) Spectroscopy

FTIR spectra of blank liposomes, CH, LF, AST-Lip, CH-AST-Lip, and LF-AST-Lip were recorded using an infrared spectrophotometer as described previously [21]. Samples were prepared via potassium bromide (KBr) tableting. KBr was placed in an oven overnight and all the samples were vacuum freeze-dried prior to analysis. Then the sample and KBr were mixed at a weight ratio of 1:100, ground into a uniform powder using a mortar and finally tableted. The scanning range used was 4000 to 400 cm^−1^ with 64 scans and resolution was set at 4 cm^−1^.

### 3.7. Measurement of DPPH Radical-Scavenging Activity

The antioxidant activity of the modified liposome was evaluated using DPPH radical scavenging capacity assay according to a reported method [22] as follows: 2 mL of ethanolic DPPH solution (0.125 mM) was mixed with 1 mL of astaxanthin liposomes or astaxanthin suspension. The mixtures were incubated for 30 min at 25 °C in the dark and then the residual DPPH concentration was determined at 517 nm using a 1240 UV-Vis spectrophotometer (Shimadzu, Kyoto, Japan). The DPPH radical-scavenging rate was evaluated by Equation (2):

(2) $$R\;=\;1-\left[\frac{\left(A-C\right)}{B}\right]\times 100\%$$

where *R* is DPPH radical-scavenging rate, %. *A* is the absorbance of the samples. *B* is the absorbance of blank samples (1 mL of water with 2 mL of DPPH ethanolic solution). C is the absorbance of the control solution (prepared by adding ethanol instead of DPPH· solution).

### 3.8. Evaluation of Thermal Stability and Storage Stability

The stability of the astaxanthin liposomes dispersed in different environments was evaluated by measuring the absorption value at 473 nm. For the determination of thermal stability, the freshly prepared samples were placed at 4, 20, 37, 55 and 70 ° C for 30 min, then cooled to room temperature in ice water immediately before measurement.

For the determination of storage stability, freshly prepared samples were stored in the dark at 4 ° C for 2, 4, 6 and 8 days. After the treatments, the amount of astaxanthin retained in the sample was determined. The retention rate of astaxanthin was calculated using Equation (3):

(3) $$R=\frac{A}{B}\times 100\%$$

where R is retention rate of astaxanthin (%), A is mount of astaxanthin retained after the treatment and B is amount of astaxanthin in the original sample.

### 3.9. Change in Size Distribution and Zeta-Potential during Storage

The prepared liposomes were sealed and stored at room temperature, and the size distribution and zeta-potential change of the samples were determined periodically during storage.

### 3.10. Determination of Bioaccessibility

To investigate the bioaccessibility of astaxanthin, the in vitro digestion of astaxanthin was investigated as previously described using a simulated model consisting of the mouth, stomach, and intestine phases [23]. The experiment was conducted at 37 ° C and the solution was continuously mixed by magnetic stirring. Artificial oral solution (ASSS) was firstly prepared and 20 mL of ASSS and 0.6 g of mucin were mixed and stirred overnight using a magnetic stirrer to prepare a simulated oral working solution (ASWS). Then 5 mL of the sample was mixed with 5 mL of ASWS and the value of pH was adjusted to 6.8. The mixture was incubated at 37 °C for 10 min. Then it was uniformly mixed with 45 mL of physiological saline and the value of pH was adjusted to 2.0 using hydrochloric acid with 3.35 mL of 3.2 mg/mL pepsin solution mixed for 1 h. After that, the value of pH was adjusted to 7.5 using 1 mol/L NaOH, 4.5 mL of the intestinal digestive enzyme (containing 4.76 mg/mL trypsin and 5.16 mg/mL cholate) was added to simulate intestinal digestion for 2 h. During the incubation, the pH of the system was maintained at 7.5 using 0.1 mol/mL NaOH solution.

A certain amount of the digested samples was centrifuged at 10,000 r/min for 30 min at 4 °C. The sample was divided into two parts, the upper layer was the micelle layer containing astaxanthin, and the lower layer containing the undigested sample, bile salt, and various dense insolubles. The bioaccessibility of astaxanthin after digestion was measured by detecting the content of astaxanthin in the supernatant, which is evaluated by Equation (4):

(4) $$B=\frac{m_{1}}{m_{2}}\times 100\%$$

where *B* is bioaccessibility of astaxanthin, %; *m_1_* is content of astaxanthin in micelles, mg; *m_2_* is content of astaxanthin in samples, mg.

### 3.11. Statistical Analyses

All experiments were performed three times, and all results are expressed as the mean ± standard deviation. Data were analyzed by analysis of variance (ANOVA) using the SPSS 18.0 software (SPSS Inc., Chicago, IL, USA). The difference between the values was determined based on Duncan’s multiple comparison test at the 5% significance level.

## 4. Conclusions

CH and LF-modified astaxanthin liposomes were successfully prepared by decorating conventional liposome formulations. The results showed that CH and LF can coat on the surface of liposome by electrostatic interaction to fill the gap of phospholipid molecules, which enhanced the physicochemical properties and stability of CH-AST-Lip and LF-AST-Lip. Compared with CH, LF exhibited a better effect on maintaining the stability of the phospholipid bilayer membrane and inhibiting the degradation of astaxanthin. The results are helpful for developing liposomes as a nutrient delivery system in the food industry, but the uptake and transport of astaxanthin by liposome modified at the cellular level and the regulation of absorption need further research and discussion.
